# Supplementary material for: Trajectories of posttraumatic growth and posttraumatic depreciation: A one-year prospective study among people living with HIV
Source: PLoS One. 2022 Sep 22;17(9):e0275000. doi: 10.1371/journal.pone.0275000 (PMC9498953; doi:10.1371/journal.pone.0275000)
Supplement: Attachment — Submitted filename: Response to Reviewers.doc [file pone.0275000.s002.doc]

**Dear Editor, Dear Reviewer,**

**thank you very much for suggestions and remarks concerning our article titled “Trajectories of posttraumatic growth and posttraumatic depreciation: A one-year prospective study among people living with HIV”, which we would like to publish in *PLOS ONE*. We have introduced all remarks mentioned in the review. Below we cite the specific remark and, in bold and in parentheses, our answer to the Reviewer’s remarks. All the changes in the manuscript were tracked and marked in yellow.**

1. Is the manuscript technically sound, and do the data support the conclusions?

The manuscript must describe a technically sound piece of scientific research with data that supports the conclusions. Experiments must have been conducted rigorously, with appropriate controls, replication, and sample sizes. The conclusions must be drawn appropriately based on the data presented.

Reviewer #1: Yes

**[Thank you very much for approval of our reasoning in this manuscript.]**

2. Has the statistical analysis been performed appropriately and rigorously?

Reviewer #1: Yes

**[Thank you very much for analysing and approving the statistical methods we chose.]**

3. Have the authors made all data underlying the findings in their manuscript fully available?

The [PLOS Data policy](http://www.plosone.org/static/policies.action#sharing) requires authors to make all data underlying the findings described in their manuscript fully available without restriction, with rare exception (please refer to the Data Availability Statement in the manuscript PDF file). The data should be provided as part of the manuscript or its supporting information, or deposited to a public repository. For example, in addition to summary statistics, the data points behind means, medians and variance measures should be available. If there are restrictions on publicly sharing data—e.g. participant privacy or use of data from a third party—those must be specified.

Reviewer #1: No

**[Thank you for this remark. We provided an anonymized dataset with our revision. All additional data regarding our datasets will be made available upon request.]**

4. Is the manuscript presented in an intelligible fashion and written in standard English?

PLOS ONE does not copyedit accepted manuscripts, so the language in submitted articles must be clear, correct, and unambiguous. Any typographical or grammatical errors should be corrected at revision, so please note any specific errors here.

Reviewer #1: Yes

**[Thank you for approving our language. The manuscript was professionally proofread.]**

5. Review Comments to the Author

Please use the space provided to explain your answers to the questions above. You may also include additional comments for the author, including concerns about dual publication, research ethics, or publication ethics. (Please upload your review as an attachment if it exceeds 20,000 characters)

Reviewer #1: General comment
The present study aimed to identify the different trajectories of posttraumatic growth (PTG) and posttraumatic depreciation (PTD) constructs and their possible predictors in a group of people living with HIV (PLWH). In order to reach this goal, a longitudinal study was carried out with three evaluation times (baseline, 6 months later, and 1 year later).
The paper deals with a clinically interesting topic, given the various trajectories that PTG and PTD may assume over time, based on different individual characteristics. However, I have some specific comments for improvement of the article that are reported below.

Specific comments:
1) In the introductive section, I would suggest the authors to include a definition of both PTG and PTD dimensions, in order to make those constructs clearer to the reader.

**[Thank you for this remark. We provided more exhaustive definitions of the two phenomena.]**

Moreover, I would suggest the authors to dedicate more space to the presentation of the available data on PTG/PTD in people leaving with HIV, reducing perhaps the first part of this section. Indeed, the association between PTG and individual characteristics is immediately exposed, without introducing this topic in the specific context of HIV.

**[Thank you for this point. We elaborated on PTG phenomenon across studies concerning PLWH.]**

Finally, in this section have been presented some methodological aspects that could be only mentioned here and deepened in the next sections of the manuscript. Similarly, the importance of assessing specific psychological factors in the context of HIV (i.e., resilience and resources) could be better highlighted.

**[Thank you for this remark. We elaborated more on the importance of assessing resilience and resources in the context of HIV.]**

2) With regard to the study aim, I would suggest the authors to integrate this subparagraph with the main body of the introduction, in order to make this section easier to be followed.

**[Thank you for this remark. We merged the two sections.]**

3) In the participants’ description, I would suggest the authors to describe the main reasons of drop-out rate. Moreover, the time period in which the evaluation has been carried out could be included.

**[Thank you for this point. We added information on possible drop out reasons and study period.]**

4) With regard to the measures administered in the present study, it is not clear if the authors employed an ad hoc Polish translation of each questionnaire or if validated and published Polish versions of those instruments are available.

**[Thank you for this point. We clarified the tool description.]**

5) I am not an expert of latent class growth analysis; however, the analyses seem to be adequately performed. I would only suggest the authors to include some sub-paragraphs (in the result section) in which the main results of the study are reported in a more organized way.

**[Thank you very much for this comment. We not only restructured the result section, but also included a clearer description of LCGA in the data analysis section.]**

6) Similar to the result section, I would suggest that the authors better organize the discussion of the main findings of the study (e.g., the following sentence “... and within both we found that resilience and resource gains and losses predicted their paths that we will discuss further on” could be confusing for the reader).

**[Thank you for this point. We appreciate your comment on this section. According to your suggestion, to make our discussion clearer to the reader we restructured the section.]**

In addition, findings could be better integrated and discussed in relation to the previous evidence that investigated the present psychological constructs both in people with HIV and other medical conditions.

**[Thank you for this remarked. We clarified and broadened our discussion in the context of previous findings.]**

7) In the conclusive section of the manuscript, I would suggest the authors to include some clinical implications of the present findings.

**[Thank you very much for this remark. We highlighted clinical, as well as wider social implication of our study.]**

8) Please correct some typing errors throughout the manuscript.

**[Thank you very much for this point. We did our best to find and correct the errors.]**

6. PLOS authors have the option to publish the peer review history of their article ([what does this mean?](https://journals.plos.org/plosone/s/editorial-and-peer-review-process#loc-peer-review-history)). If published, this will include your full peer review and any attached files.


If you choose “no”, your identity will remain anonymous but your review may still be made public.


**Do you want your identity to be public for this peer review?** For information about this choice, including consent withdrawal, please see our [Privacy Policy](https://www.plos.org/privacy-policy).

Reviewer #1: No
